# Supplementary figures and images for: Synapsin E-domain is essential for α-synuclein function
Source: eLife. 2024 May 7;12:RP89687. doi: 10.7554/eLife.89687 (PMC11076041; doi:10.7554/eLife.89687)

Western blot F2B

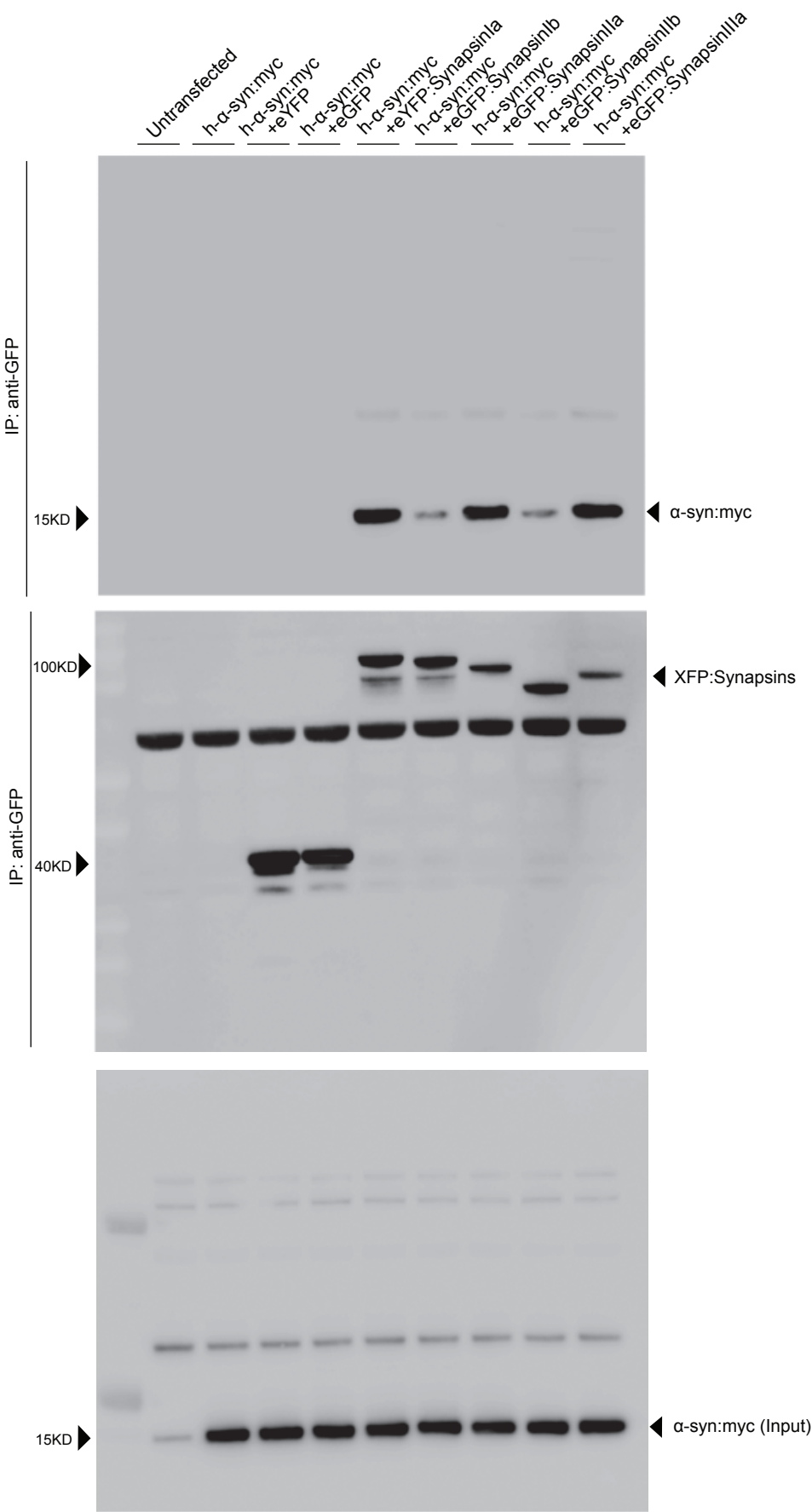

Supplement: Figure 2—source data 2. [file elife-89687-fig2-data2.pdf]

Western blot F2G

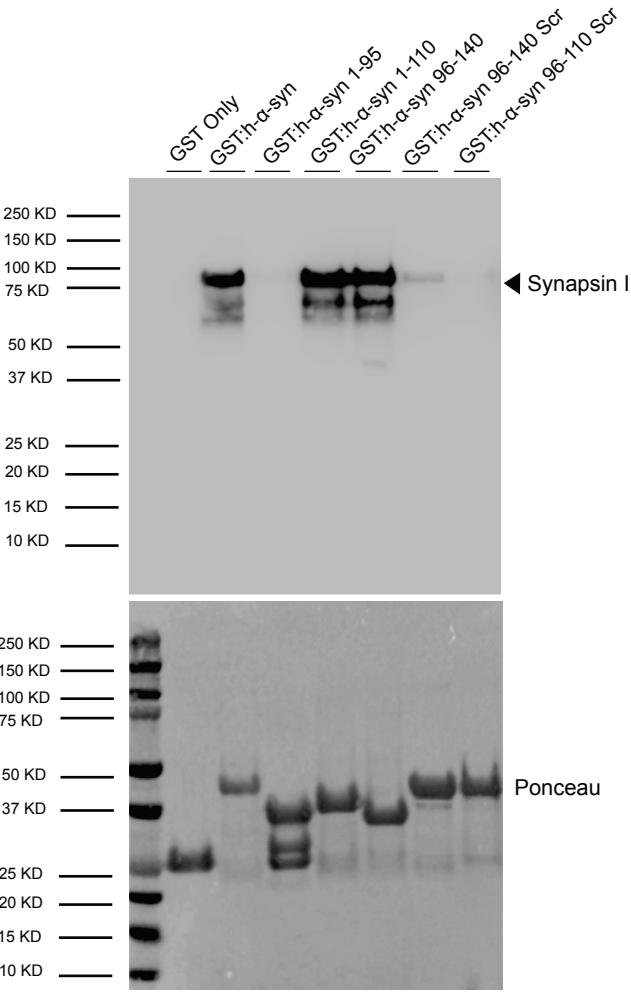

Supplement: Figure 2—source data 3. [file elife-89687-fig2-data3.pdf]

Western blot F3D

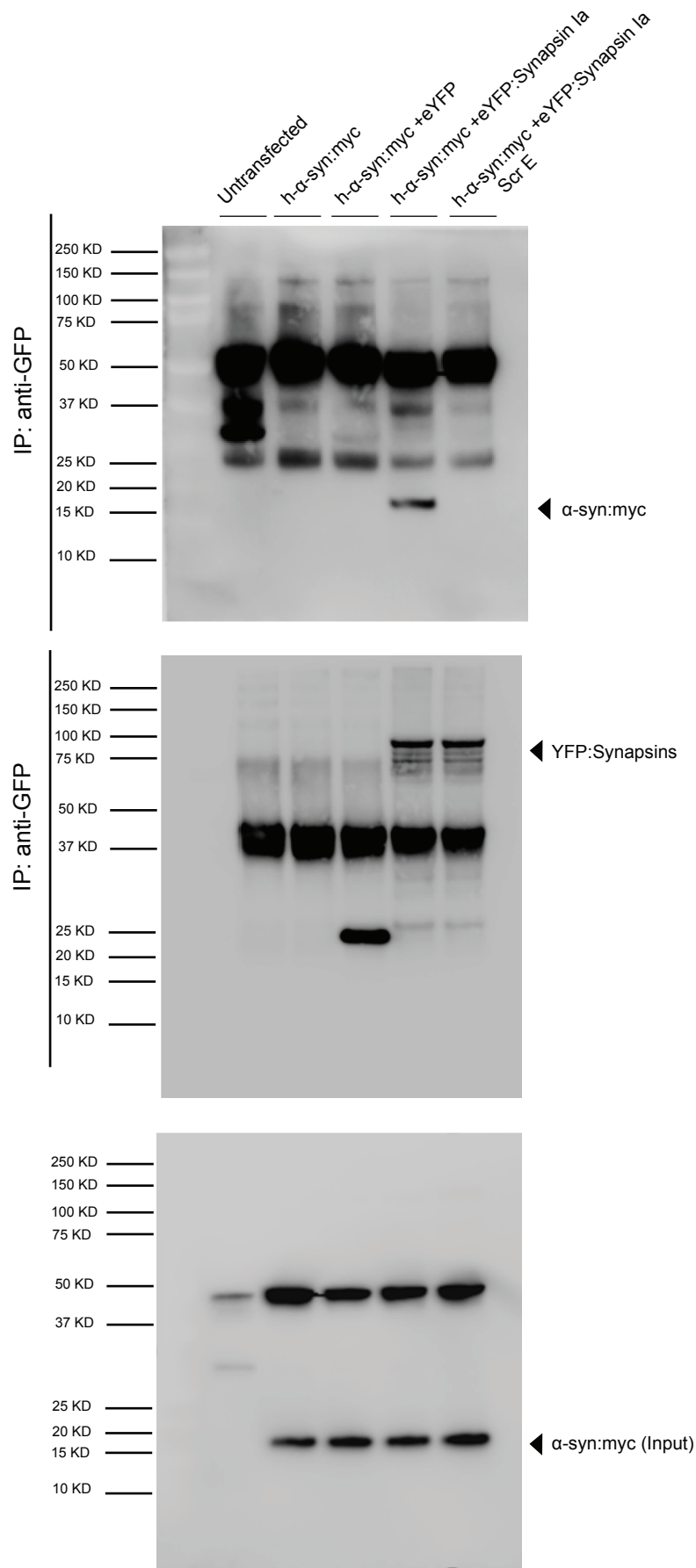

Supplement: Figure 3—source data 2. [file elife-89687-fig3-data2.pdf]

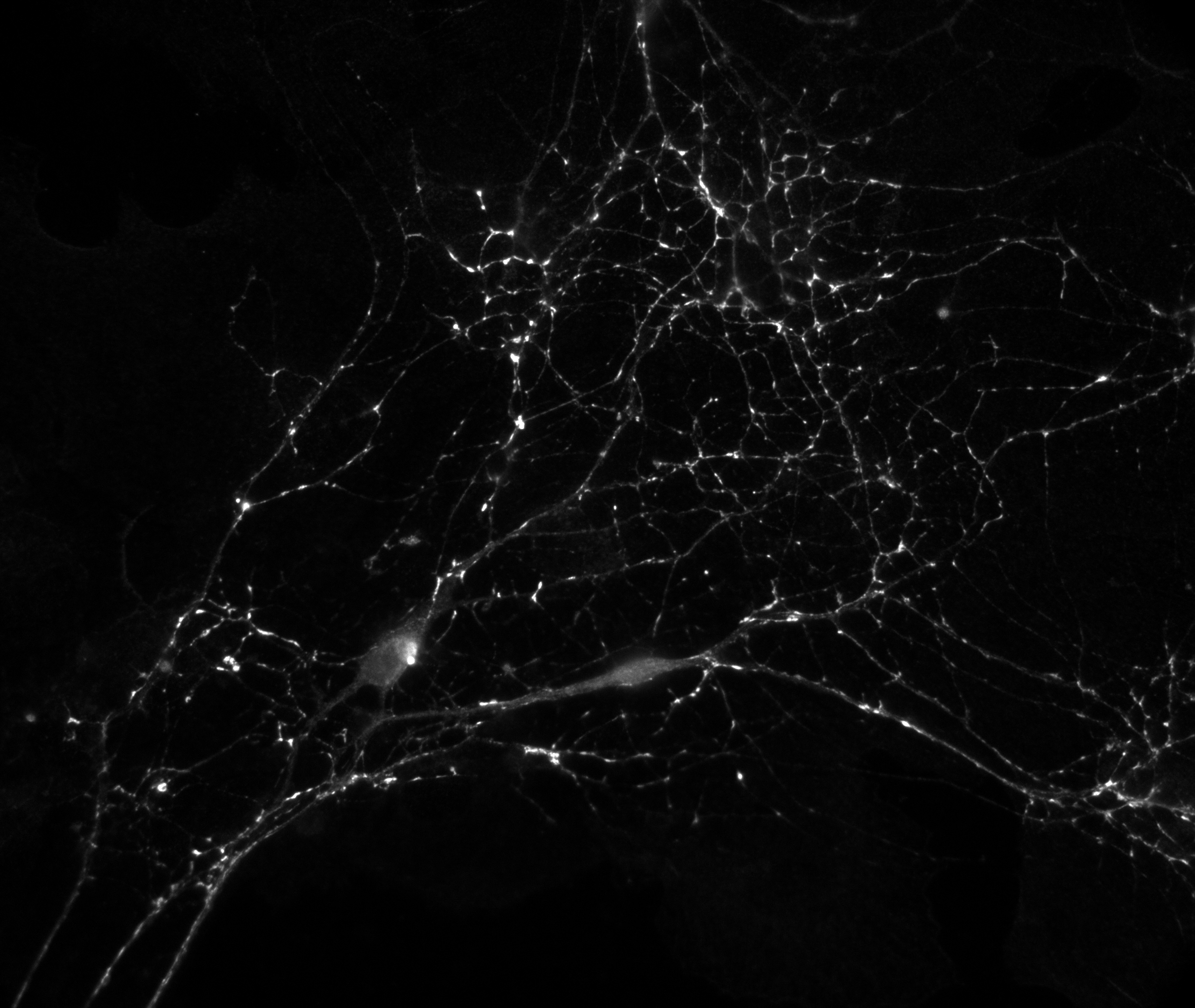

Supplement: Figure 3—figure supplement 1—source data 2. — Full images of anti-synapsin I and anti-vGlut1 immunofluorescence channels containing the details shown in panel B (marked with white dashed square). [file elife-89687-fig3-figsupp1-data2.zip › Figure 3 Suppl figure 1 whole image Synapsin Ia Synapsin I IF.tif]

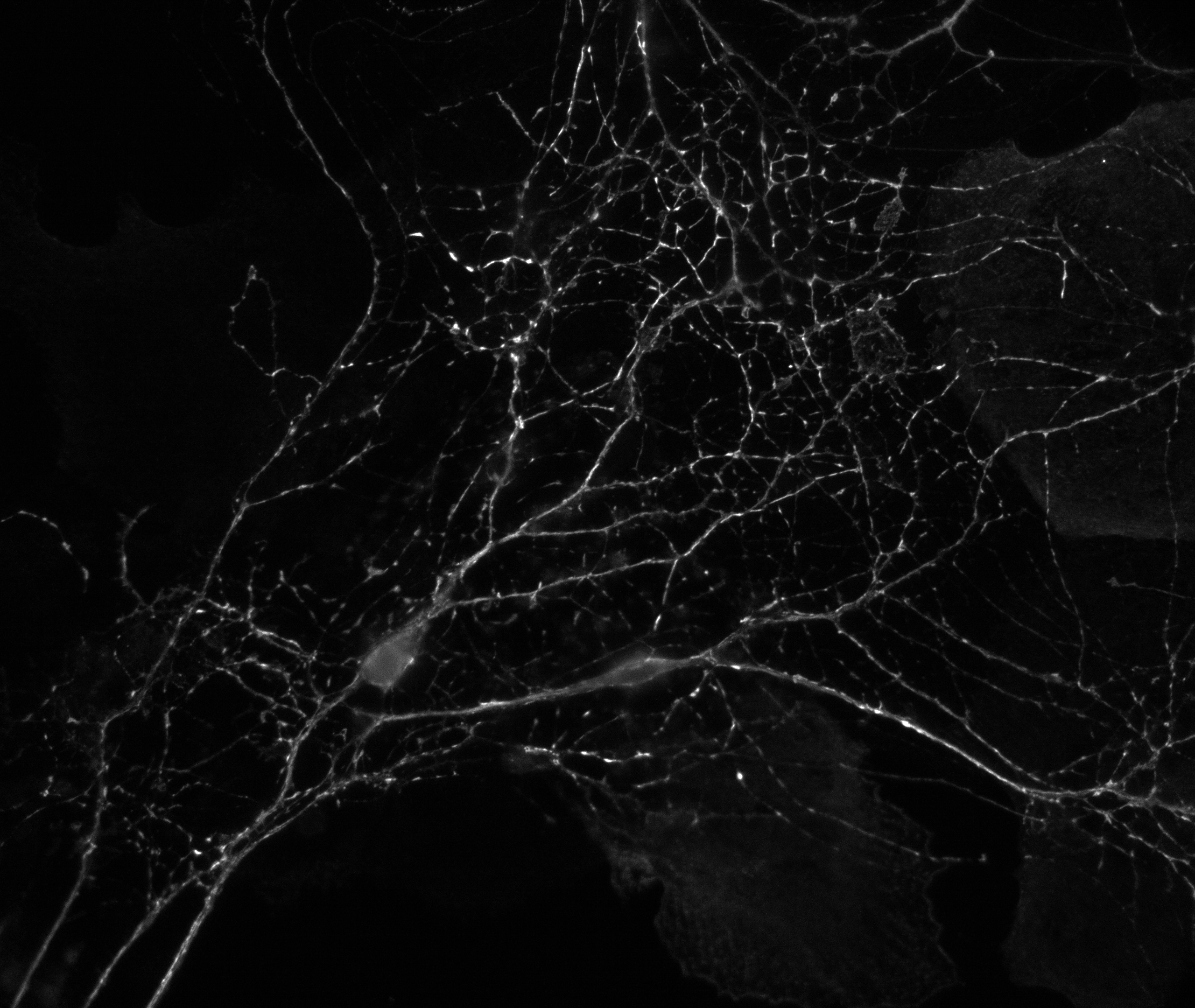

Supplement: Figure 3—figure supplement 1—source data 2. — Full images of anti-synapsin I and anti-vGlut1 immunofluorescence channels containing the details shown in panel B (marked with white dashed square). [file elife-89687-fig3-figsupp1-data2.zip › Figure 3 Suppl figure 1 whole image Synapsin Ia vGlut1 IF.tif]

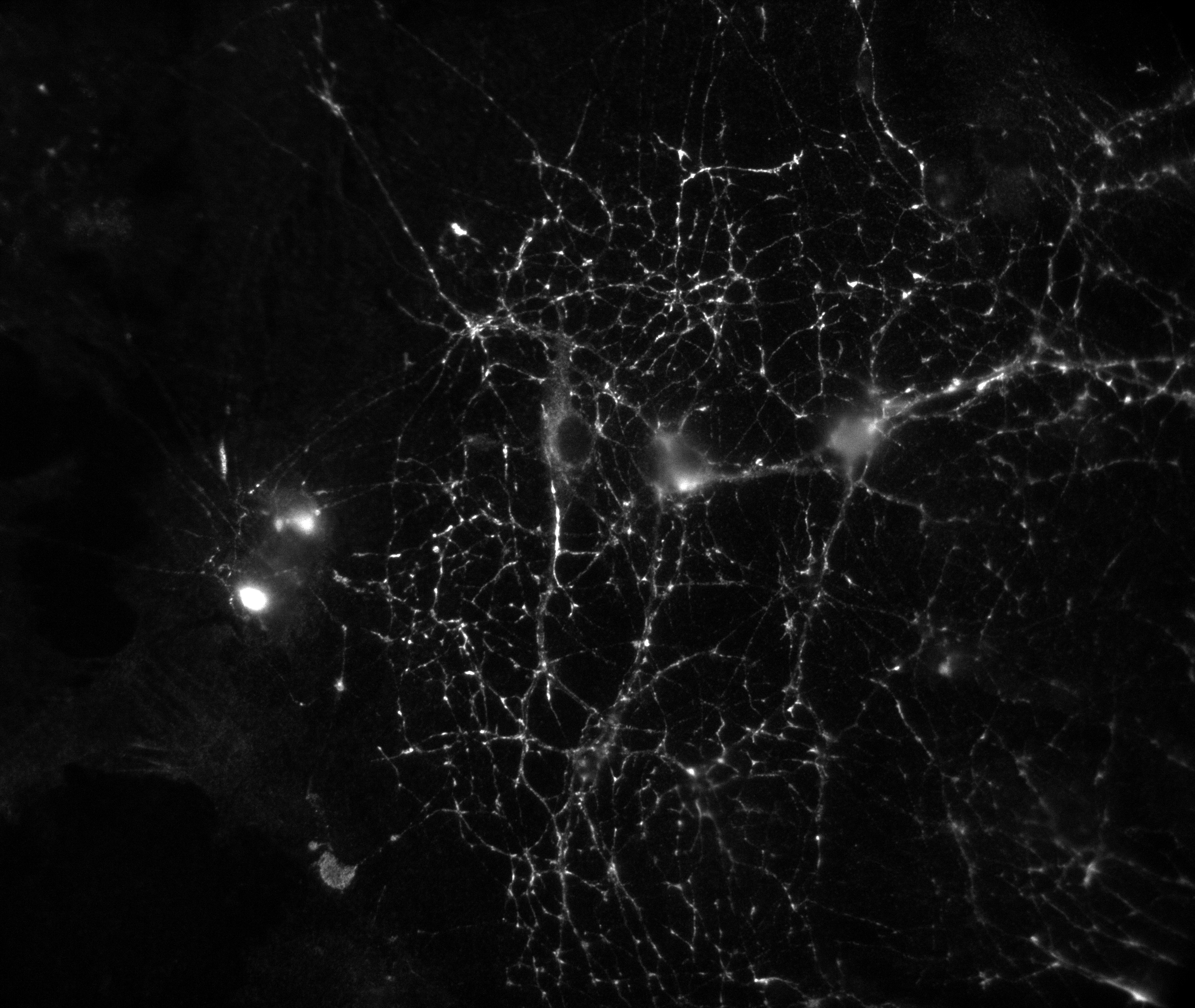

Supplement: Figure 3—figure supplement 1—source data 2. — Full images of anti-synapsin I and anti-vGlut1 immunofluorescence channels containing the details shown in panel B (marked with white dashed square). [file elife-89687-fig3-figsupp1-data2.zip › Figure 3 Suppl figure 1 whole image Synapsin IaScrE vGlut1 IF.tif]

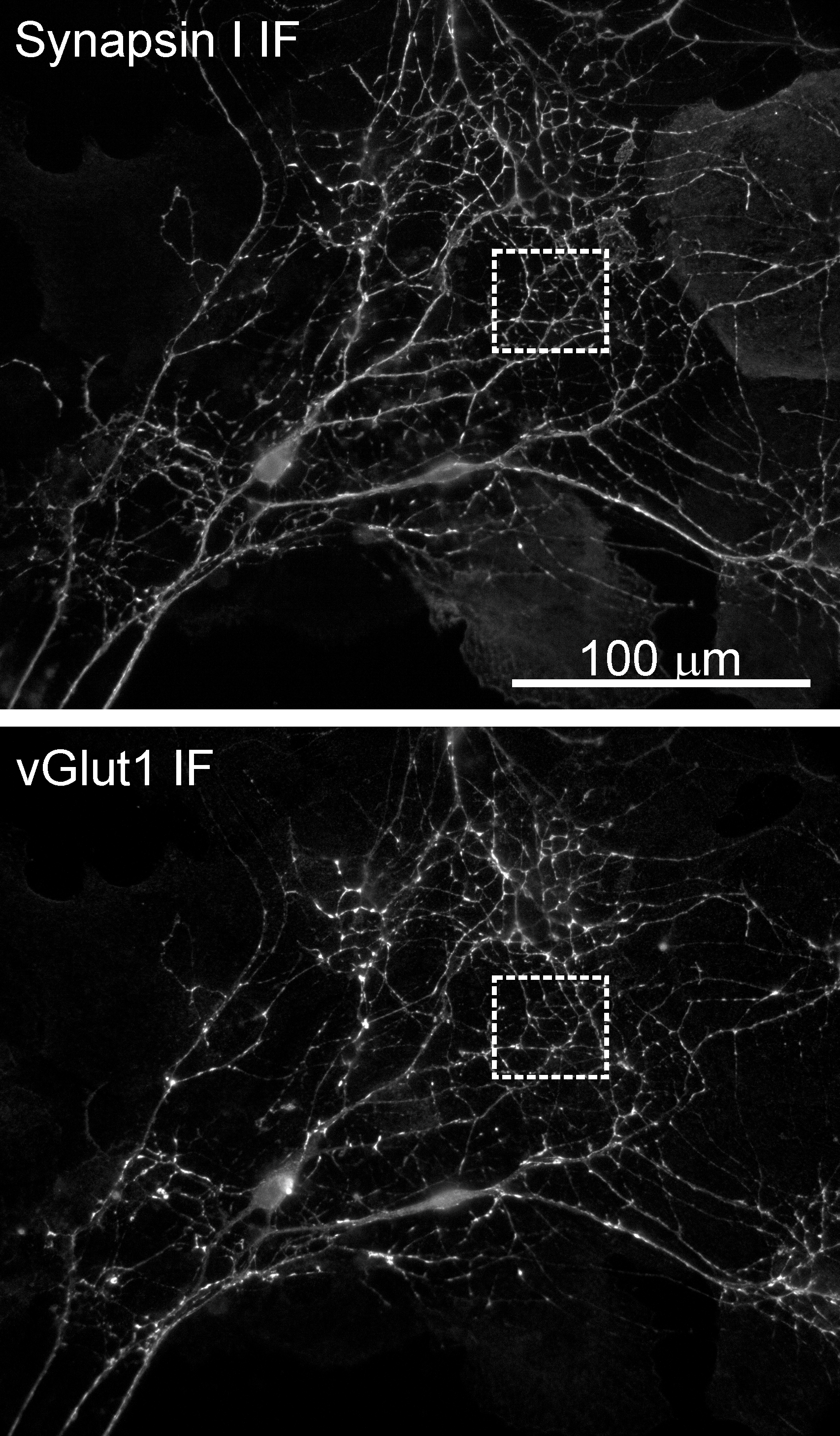

Supplement: Figure 3—figure supplement 1—source data 2. — Full images of anti-synapsin I and anti-vGlut1 immunofluorescence channels containing the details shown in panel B (marked with white dashed square). [file elife-89687-fig3-figsupp1-data2.zip › Figure 3 Suppl figure 1 whole images SynIa.tif]

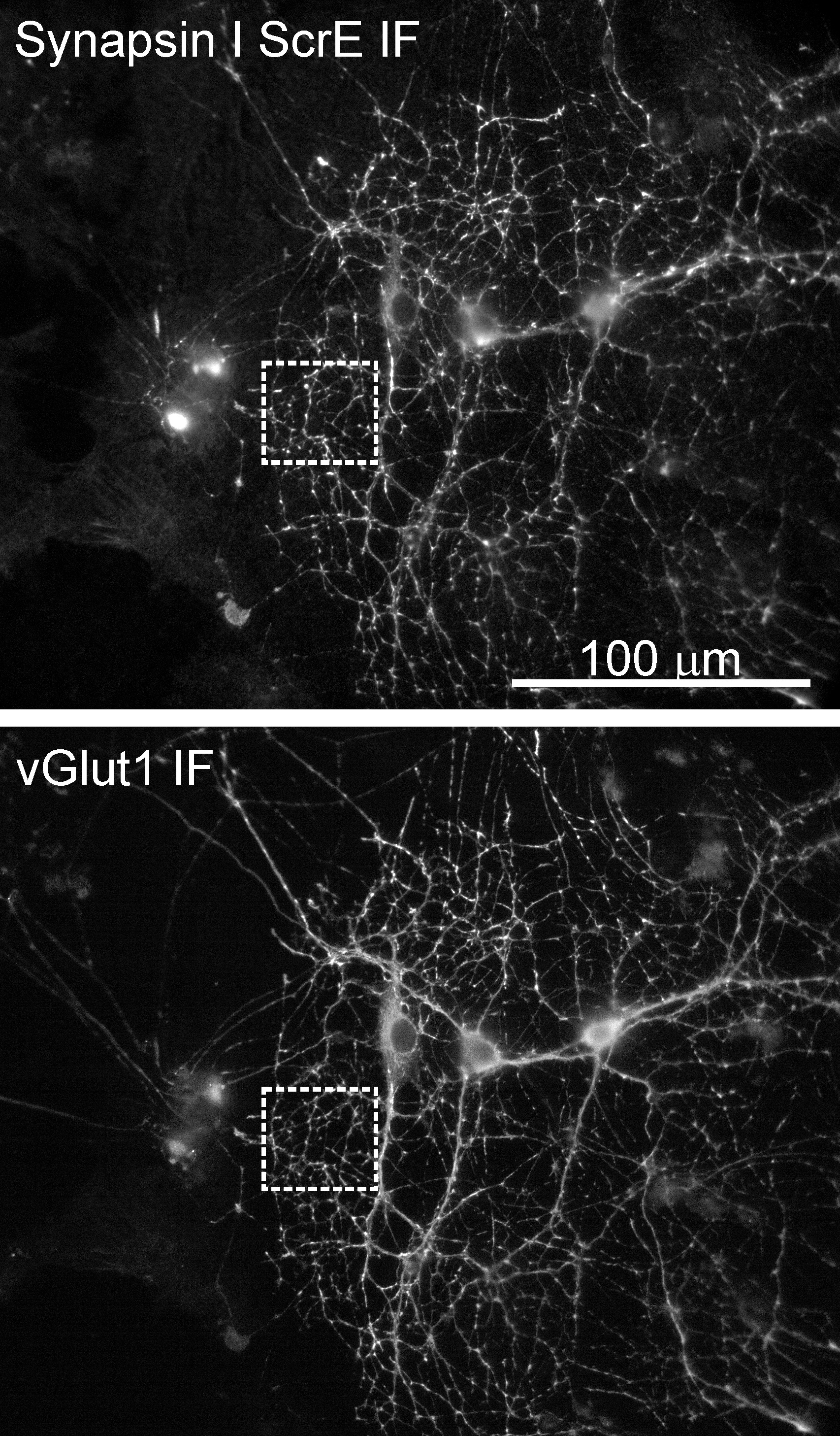

Supplement: Figure 3—figure supplement 1—source data 2. — Full images of anti-synapsin I and anti-vGlut1 immunofluorescence channels containing the details shown in panel B (marked with white dashed square). [file elife-89687-fig3-figsupp1-data2.zip › Figure 3 Suppl figure 1 whole images SynIaScrE.tif]

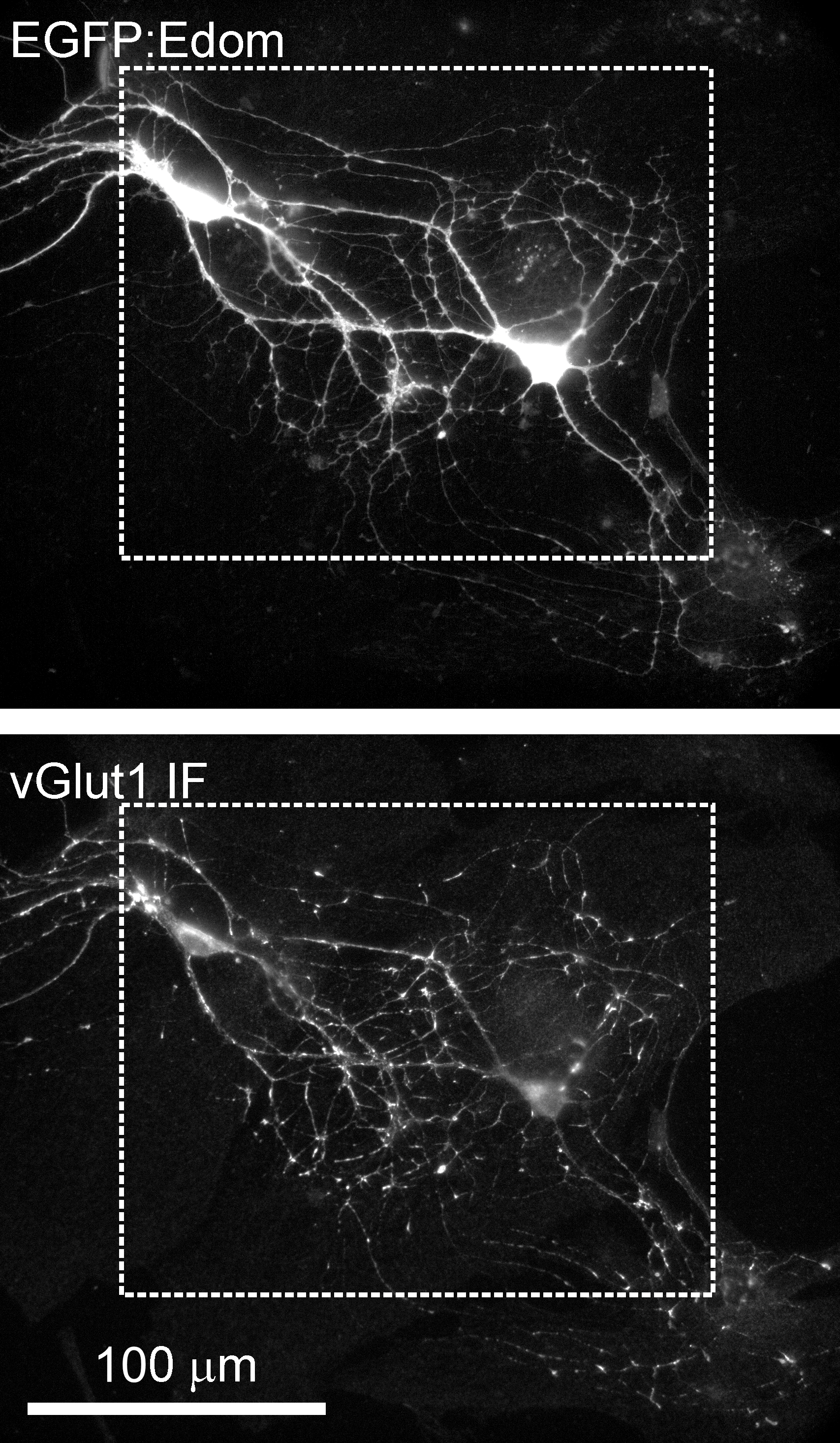

Supplement: Figure 3—figure supplement 2—source data 2. — Full images of EGFP:E-domain and anti-vGlut1 immunofluorescence channels containing the details shown in panel B, and the full images of sypHy:E-domain and anti-vGlut1 immunofluorescence channels containing the details shown in panel D (marked with white dashed square). [file elife-89687-fig3-figsupp2-data2.zip › Figure 3 Suppl figure 2 whole images EGFP-E.tif]

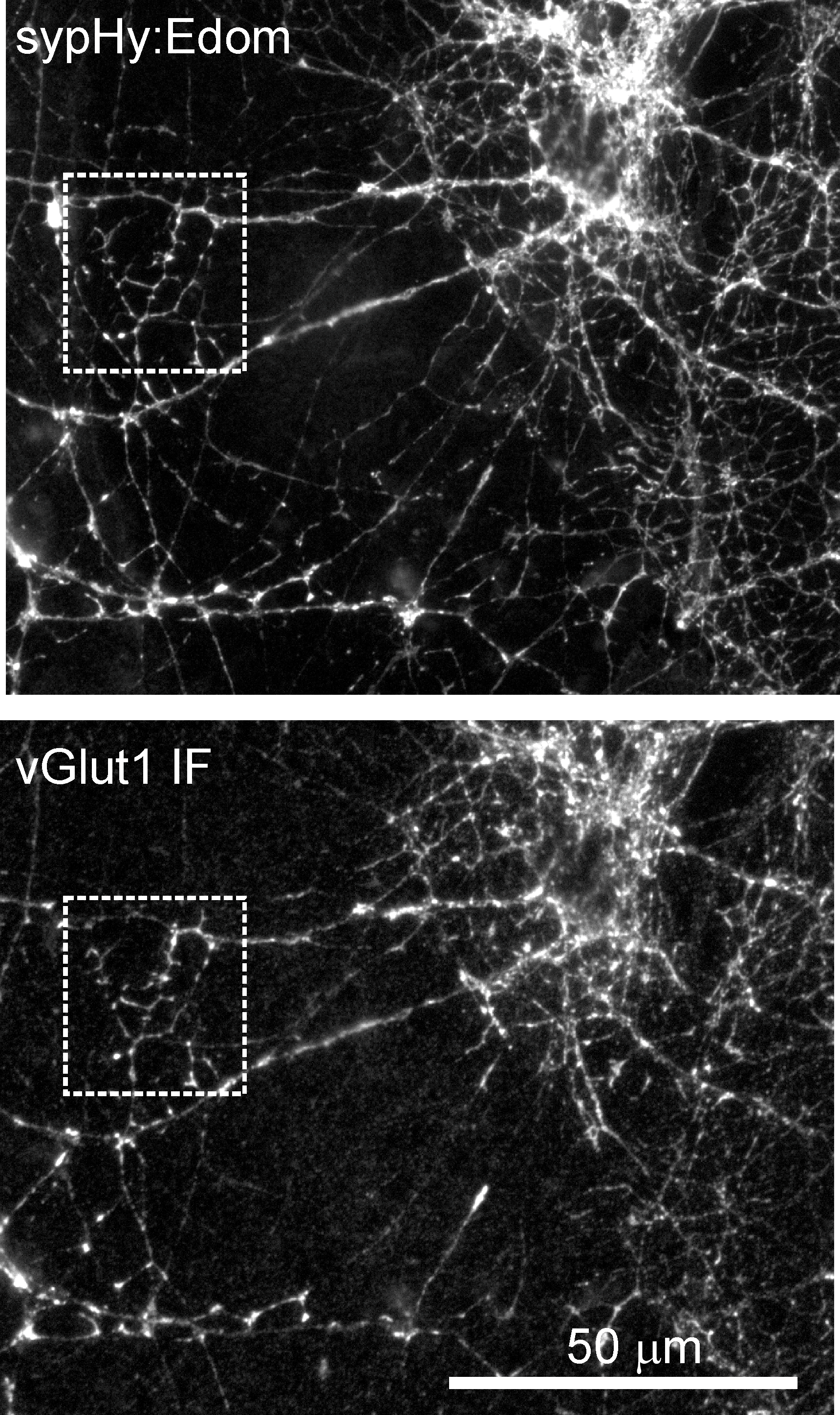

Supplement: Figure 3—figure supplement 2—source data 2. — Full images of EGFP:E-domain and anti-vGlut1 immunofluorescence channels containing the details shown in panel B, and the full images of sypHy:E-domain and anti-vGlut1 immunofluorescence channels containing the details shown in panel D (marked with white dashed square). [file elife-89687-fig3-figsupp2-data2.zip › Figure 3 Suppl figure 2 whole images sypHy-E.tif]

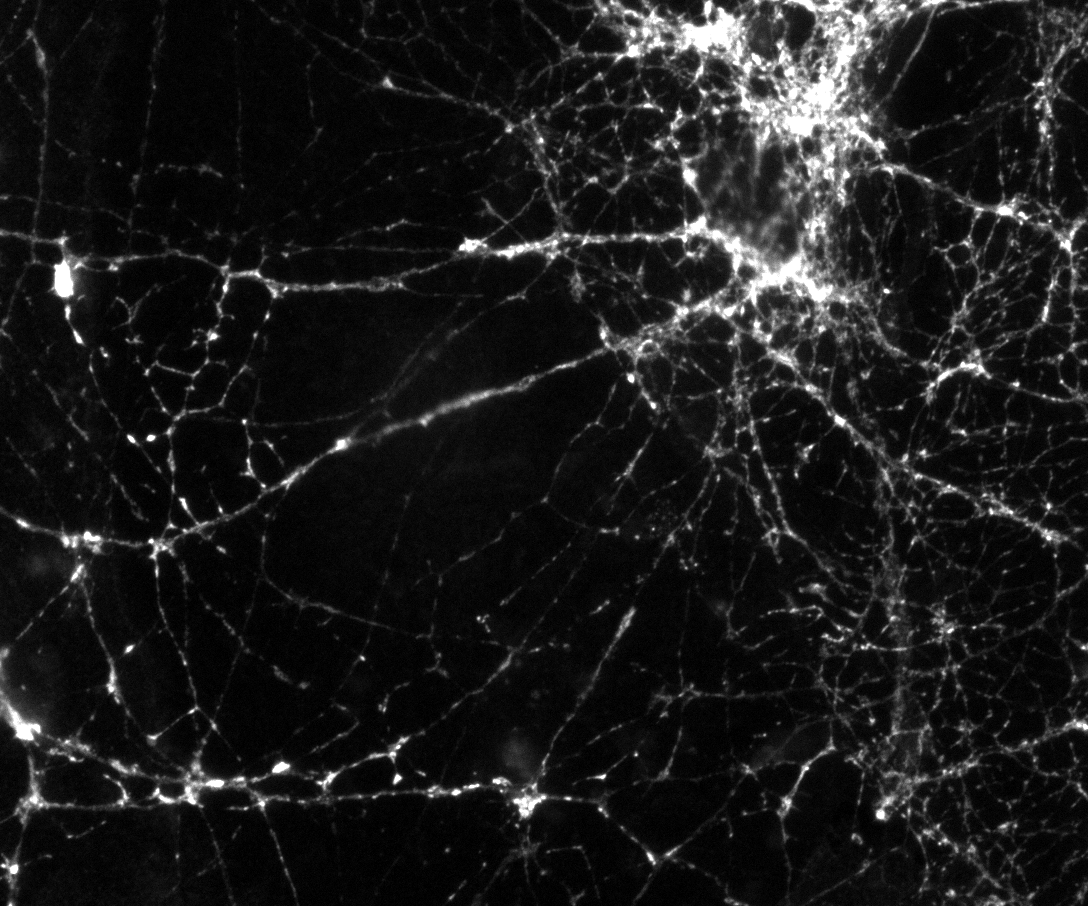

Supplement: Figure 3—figure supplement 2—source data 2. — Full images of EGFP:E-domain and anti-vGlut1 immunofluorescence channels containing the details shown in panel B, and the full images of sypHy:E-domain and anti-vGlut1 immunofluorescence channels containing the details shown in panel D (marked with white dashed square). [file elife-89687-fig3-figsupp2-data2.zip › sypHyE EGFP channel.tif]

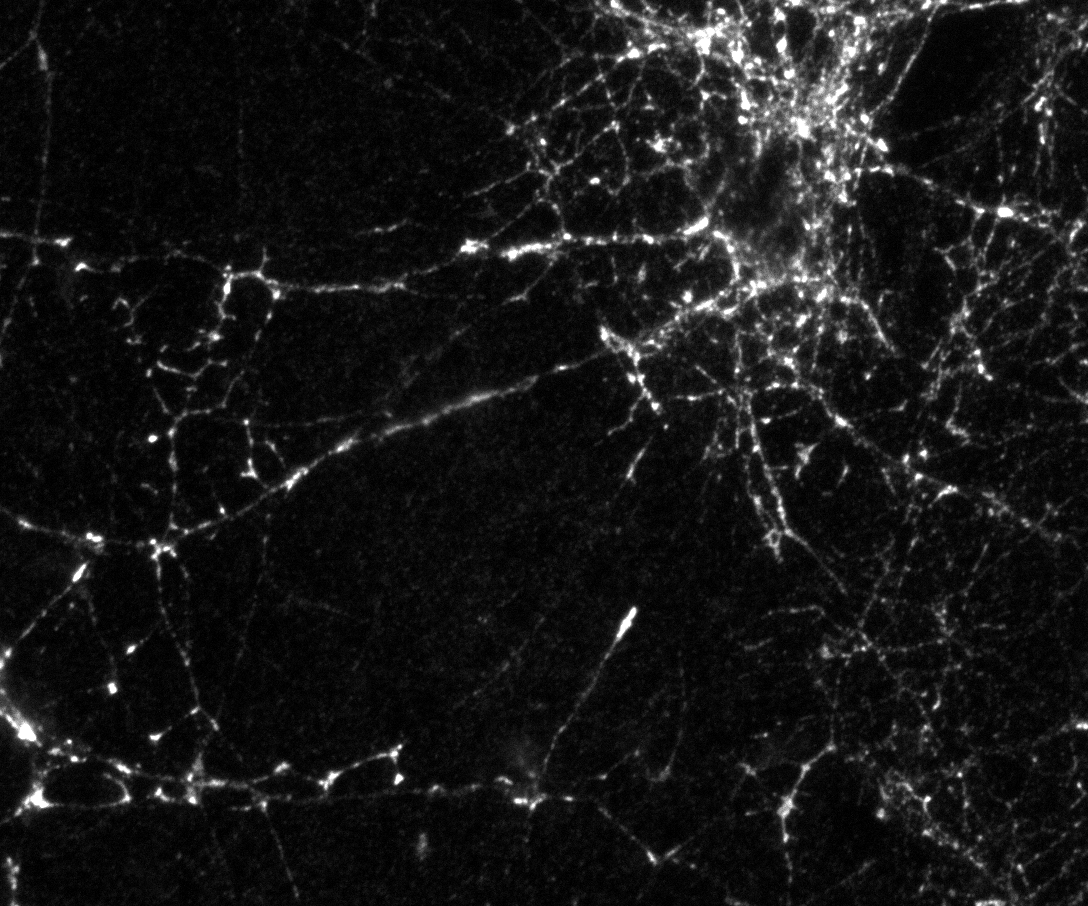

Supplement: Figure 3—figure supplement 2—source data 2. — Full images of EGFP:E-domain and anti-vGlut1 immunofluorescence channels containing the details shown in panel B, and the full images of sypHy:E-domain and anti-vGlut1 immunofluorescence channels containing the details shown in panel D (marked with white dashed square). [file elife-89687-fig3-figsupp2-data2.zip › sypHyE vGlut1 channel.tif]

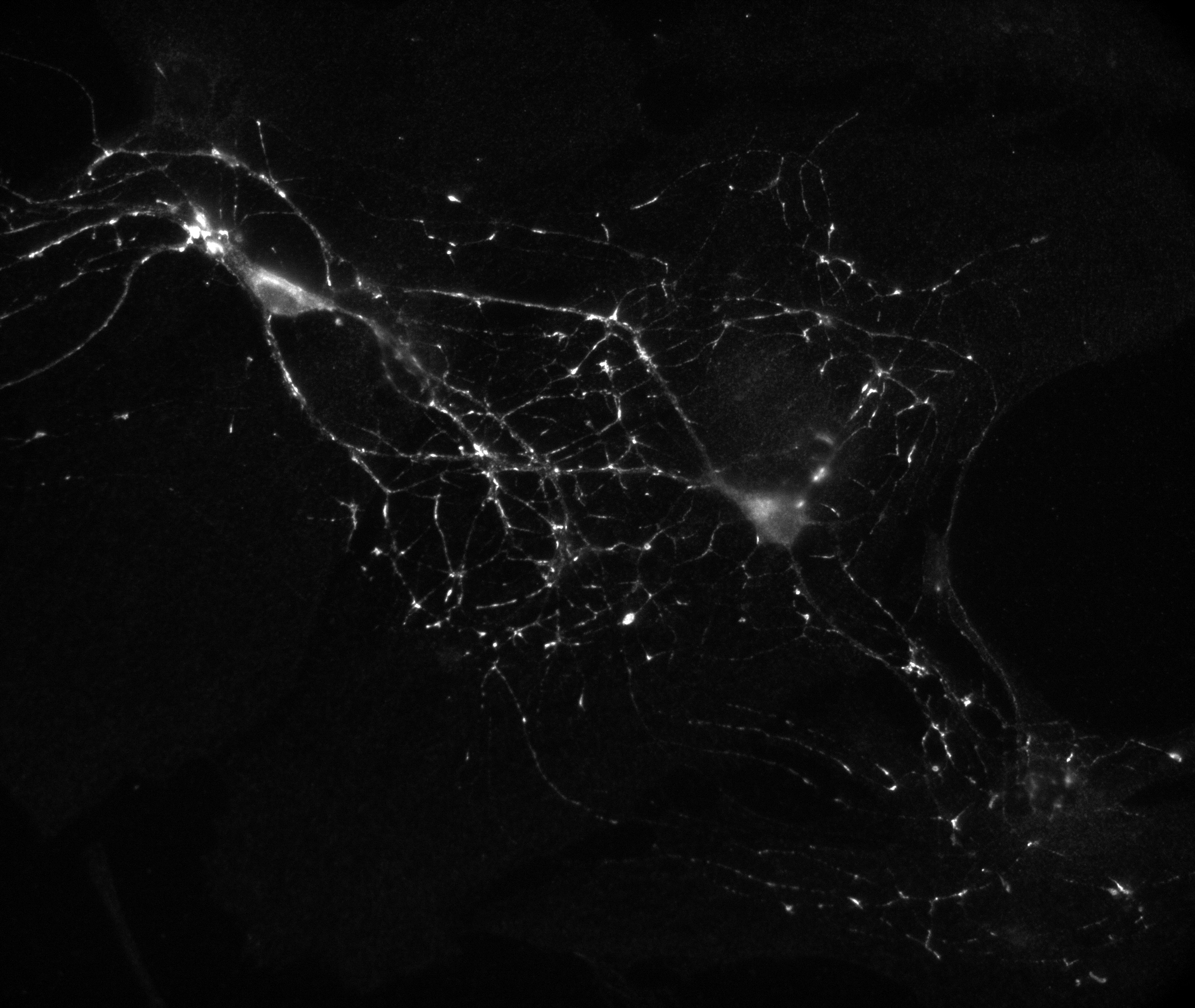

Supplement: Figure 3—figure supplement 2—source data 2. — Full images of EGFP:E-domain and anti-vGlut1 immunofluorescence channels containing the details shown in panel B, and the full images of sypHy:E-domain and anti-vGlut1 immunofluorescence channels containing the details shown in panel D (marked with white dashed square). [file elife-89687-fig3-figsupp2-data2.zip › TKO EGFP-E vGlut1.tif]

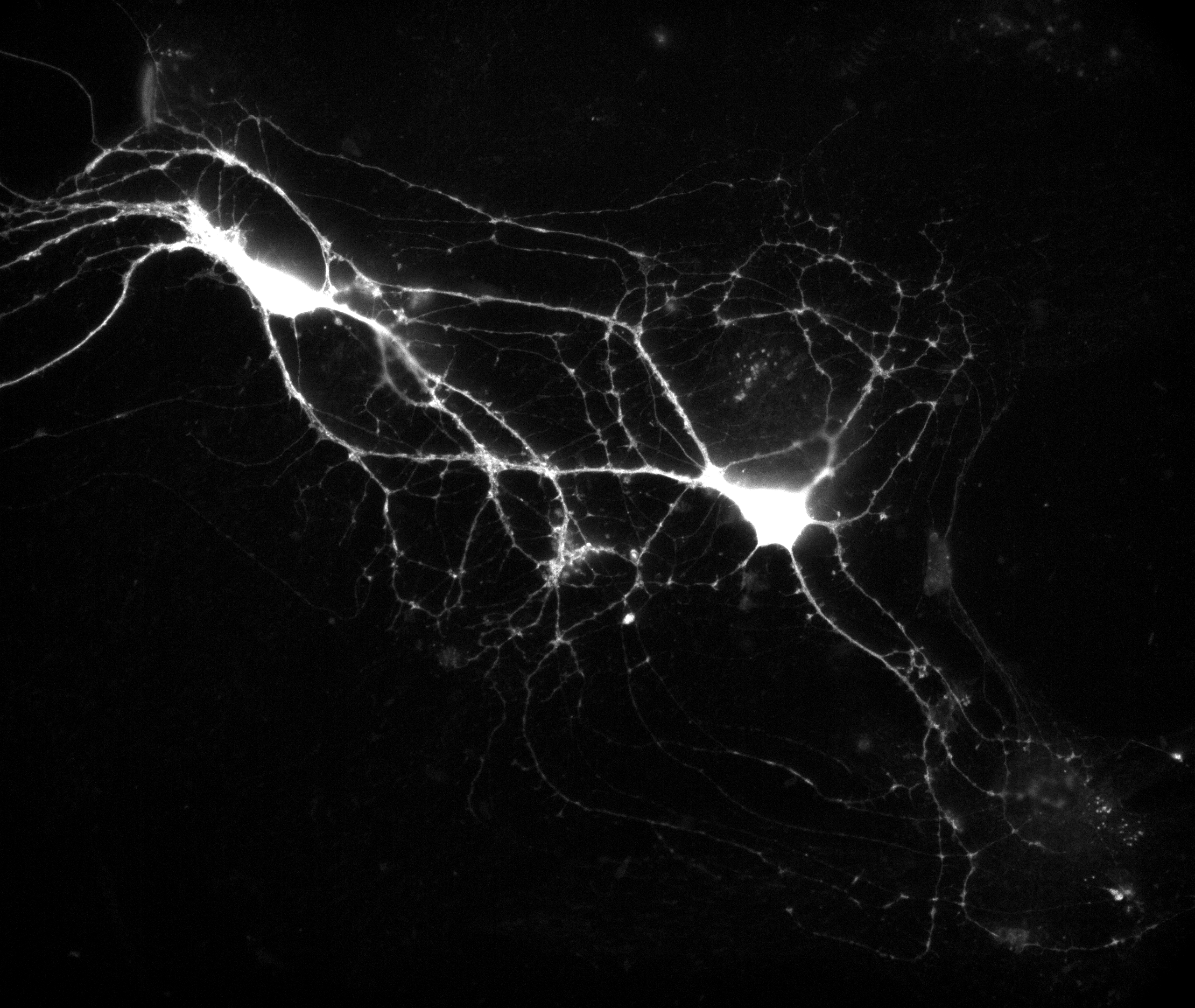

Supplement: Figure 3—figure supplement 2—source data 2. — Full images of EGFP:E-domain and anti-vGlut1 immunofluorescence channels containing the details shown in panel B, and the full images of sypHy:E-domain and anti-vGlut1 immunofluorescence channels containing the details shown in panel D (marked with white dashed square). [file elife-89687-fig3-figsupp2-data2.zip › TKO EGFP-E.tif]

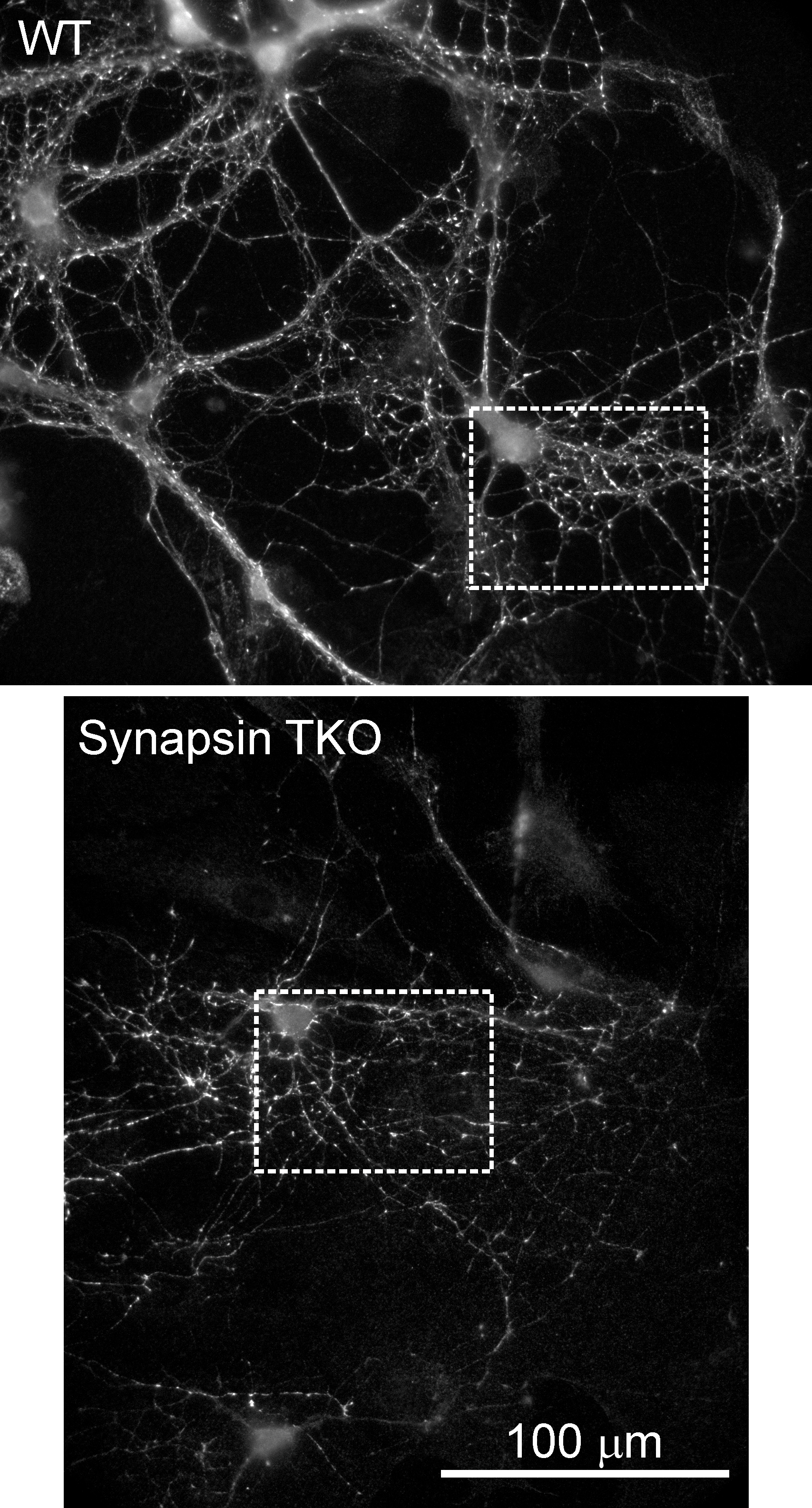

Supplement: Figure 4—source data 2. [file elife-89687-fig4-data2.zip › Figure 4 whole images.tif]

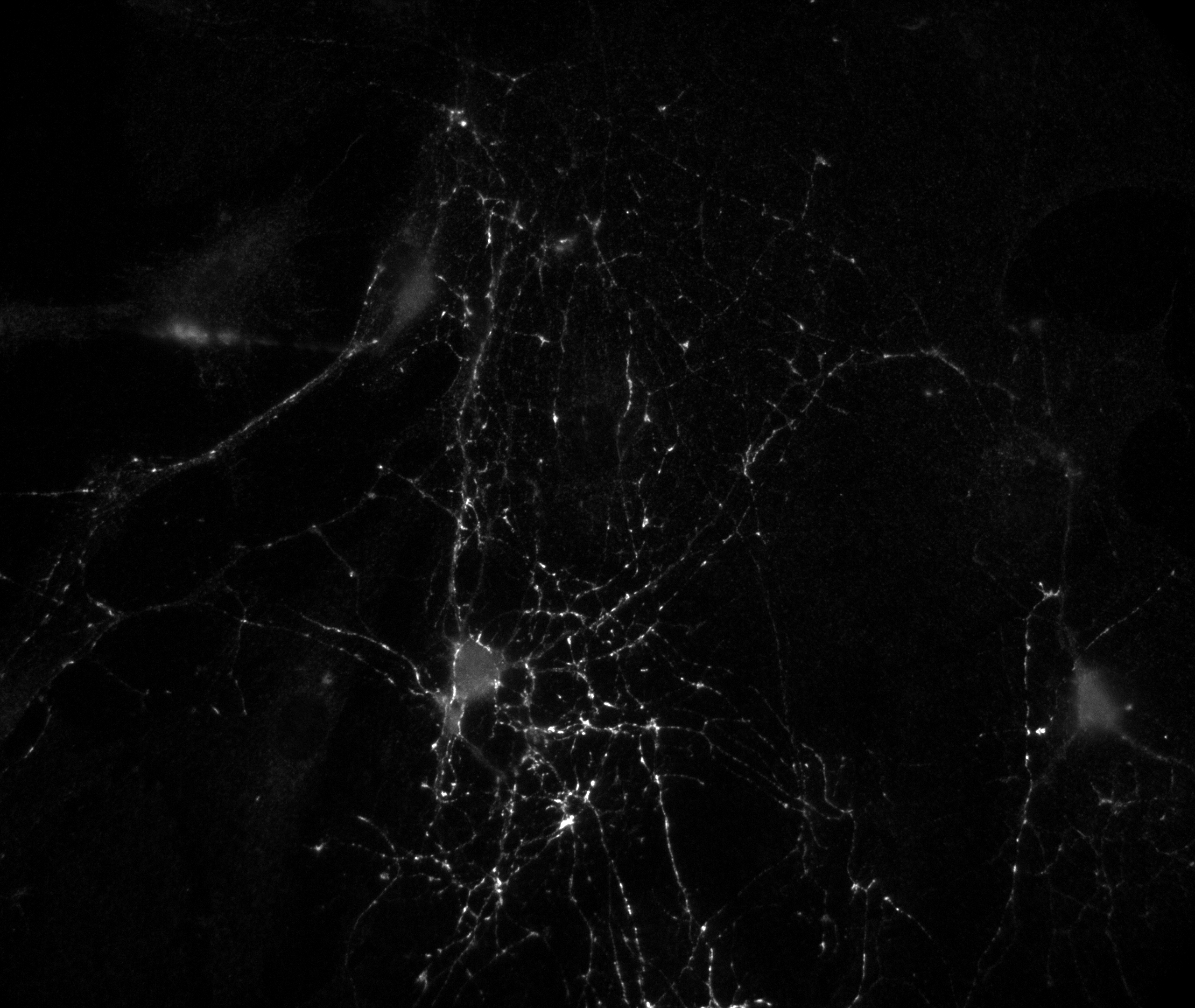

Supplement: Figure 4—source data 2. [file elife-89687-fig4-data2.zip › TKO 010.tif]

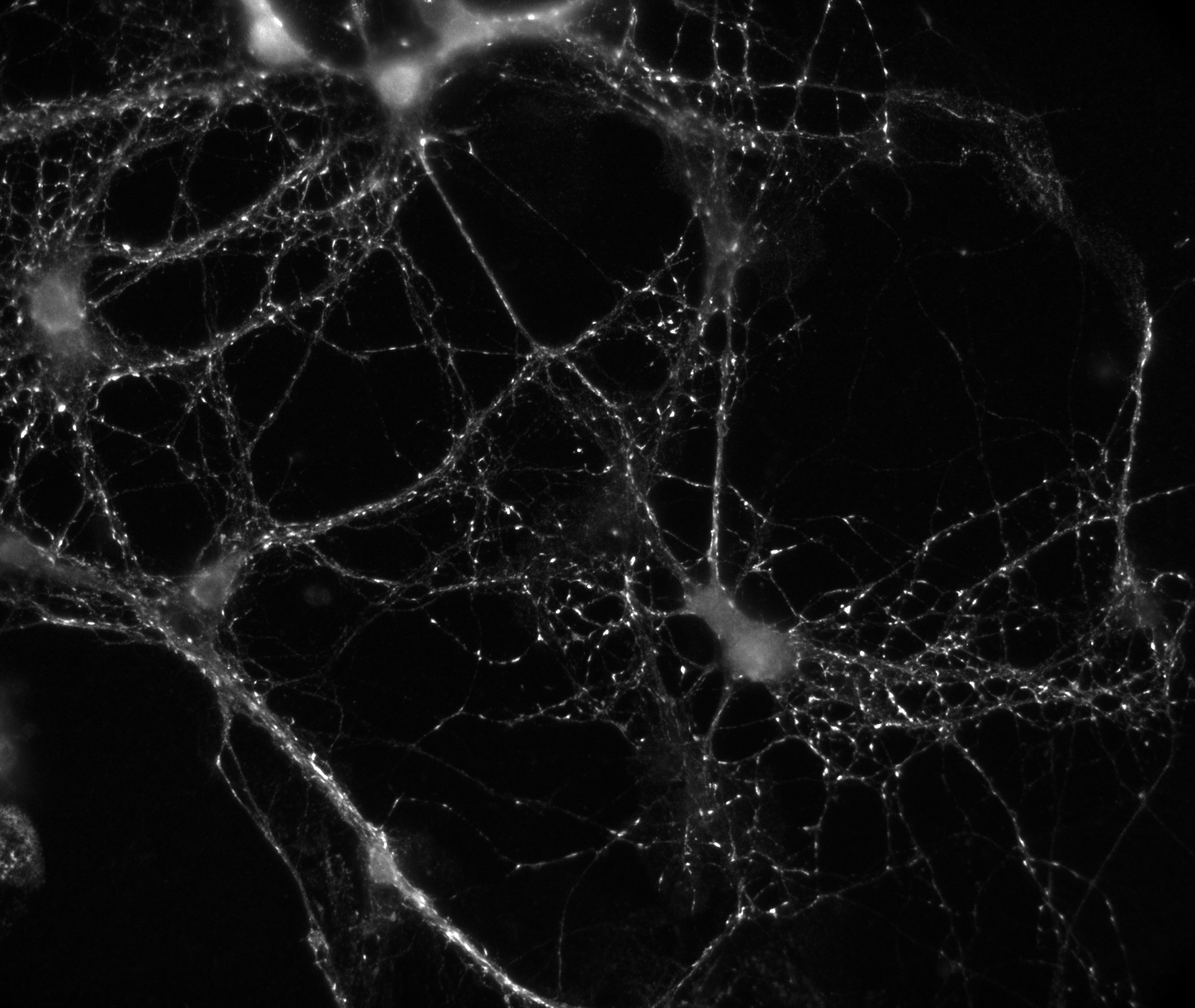

Supplement: Figure 4—source data 2. [file elife-89687-fig4-data2.zip › WT 009.tif]
